# TREATMENT OF OSTEOGENESIS IMPERFECTA USING THE FASSIER-DUVAL TELESCOPIC ROD

**DOI:** 10.1590/1413-785220233103e266775

**Published:** 2023-09-08

**Authors:** Ellen de Oliveira Goiano, Miguel Akkari, Paulo Humberto Costa, Marina Rafaele Makishi, Cláudio Santili

**Affiliations:** 1 Irmandade da Santa Casa de Misericórdia de São Paulo, Pediatric Orthopedics and Traumatology Group, Sao Paulo, SP, Brazil.; 2 Irmandade da Santa Casa de Misericórdia de São Paulo, Programa de Ortopedia e Traumatologia Pediátrica, Sao Paulo, SP, Brazil.; 3 Santa Casa de Misericórdia de São Paulo, Faculdade de Ciências Médicas, Sao Paulo, SP, Brazil.; 4 Irmandade da Santa Casa de Misericórdia de São Paulo, Department of Orthopedics and Traumatology, Sao Paulo, SP, Brazil.

**Keywords:** Osteogenesis imperfecta, Osteotomy, Joint Deformities, Acquired, Osteogênese Imperfeita, Osteotomia, Deformidades Articulares Adquiridas

## Abstract

**Objectives:**

This study aimed to assess the treatment of patients with Osteogenesis Imperfecta (OI) operated on with a telescopic Fassier-Duval (FD) rod in a querterenario hospital from 2010 to 2020.

**Methods:**

We analyzed indication for surgical treatment, causes of reoperation, complications and the effectiveness of telescoping rod.

**Results:**

The results were compared with the literature and with the same parameters from a previous study which a different telescopic rod developed by the same authors. This was a retrospective study based on the analysis of digital and radiographic clinical records. Fifteen patients with 21 FD rods were evaluated, most were used on the femur (18 rods or 85.7%), eight patients were female (53.3%), with a mean age of 10.47 (3.92 to 16.44) years, most of whom had type III Sillence (46.7%), with a mean follow-up of 5.22 (1.43 to 7.02) years. Seven rods (33.3%) had complications. The main indication was for fracture (57.1%). Regarding the ability to telescope, we observed that 15 rods (71.4%) followed the child's growth.

**Conclusion:**

We had good results using FD rods, similar to the data found in the literature and the data obtained with our rod. ***Level of Evidence III,Retrospective comparative study*** .

## RESUMO

**Objetivos:**

O objetivo deste estudo foi avaliar o tratamento de pacientes com Osteogênese Imperfeita (OI) operados com a haste telescopada de Fassier-Duval (FD) num hospital quaternário no período de 2010 a 2020.

**Métodos:**

Analisamos a indicação cirúrgica do tratamento, as causas de revisão, suas complicações e a eficácia na telescopagem da haste.

**Resultados:**

Os resultados foram comparados com a literatura e com os mesmos parâmetros de um artigo anterior no qual foi utilizada uma haste telescopada desenvolvida pelo nosso grupo. O estudo foi retrospectivo baseado na análise dos prontuários clínicos digitais e radiográficos dos pacientes. Quinze pacientes com 21 hastes de FD foram avaliados, sendo a maioria no fêmur (85,7%), oito pacientes eram do sexo feminino (53,3%), com média de 10,47 (3,92 a 16,44) anos, a maioria do tipo III de Sillence (46,7%), com tempo de seguimento médio de 5,22 (1,43 a 7,02) anos. Deste total, sete hastes (33,3%) apresentaram complicações. A principal indicação cirúrgica foram fraturas (57,1%). Em relação à telescopagem, observamos que 15 hastes (71,4%) acompanharam o crescimento da criança.

**Conclusão:**

No presente estudo verificamos bons resultados com as hastes de FD, à semelhança dos dados encontrados na literatura e dos dados encontrados com a haste do nosso serviço. ***Nível de Evidência III; Estudo retrospectivo comparativo*** .

## INTRODUCTION

Osteogenesis imperfecta (OI) is a disease characterized by quantitative and/or qualitative changes in type I collagen. These changes lead to fragility of the bone that predisposes it to deformities and a greater number of fractures in these patients, even when low-energy trauma is involved.^1 , 2^ Vitamin D replacement, calcium and especially bisphosphonates are used in the clinical treatment of moderate and severe cases, having a positive impact upon the prognosis of the disease.^3^ For its surgical treatment, the use of telescopic intramedullary rods is recommended as the gold standard.^4 – 12^

The objective of this study was to assess the effectiveness of the Fassier-Duval (FD) rod by conducting a retrospective analysis of patients with Osteogenesis Imperfecta operated by our team.

## METHODS

This is an observational, retrospective, longitudinal study in which the physical or electronic medical records and radiographic examinations of patients with OI who had undergone intramedullary alignment and stabilization with the FD rod between January 2010 and January 2020 were analyzed.

All patients with OI who had undergone surgical treatment using FD rods were included. Those patients who did not have an appropriate outpatient follow-up, for whom there were no radiographic records, and those with other bone and metabolic diseases were excluded from the study. All patients or caregivers signed a consent and/or assent form.

The follow-up visits for each patient, the immediate postoperative images following FD rodding and the latest images that were made available within the study period were evaluated. Each rod was then checked for telescoping in millimeters by comparing the rod's measurements in the immediate postoperative period and those taken from the patient's latest radiograph, by employing the digital measurement capability provided along with the software used to perform the radiographs (Enterprise ImagingXero®Viewer – AgfaHealthCare, version 8.1.2).

The current study was approved by the ethics committee.

Data were fed to two Microsoft Excel® spreadsheets. The first included the patients’ identification data, including sex, date of birth, age at the end of data analysis, OI clinical type, date of surgery using the Fassier-Duval rod, age at the time of surgery, laterality, and whether there were surgical revisions of the implants. The second spreadsheet had data for each rod, also analyzing sex, clinical type, laterality, date of surgery, surgical indication, segment undergoing surgery, follow-up time, positive or negative telescoping, and percentage of telescoping; where pertinent, rod revision surgery and indication.

The data were statistically correlated by using the Kolmogorov-Smirnov, paired T-Student and ANOVA tests, with the SPSS **V2** 0, Minitab 16 and Microsoft Excel Office 2010 programs.

## RESULTS

Seventeen patients with OI who underwent FD rodding were considered eligible for the study. However, two were excluded due to lack of outpatient follow-up and recent radiographs. Among the 15 patients included, eight (53.3%) were female and seven (46.7%) were male. An average of 1.4 rods was obtained for each patient, totaling 21 rods. Six of them had two FD rods; five had rods bilaterally in the femurs; and one patient had one rod in the left tibia and one rod in the right femur. ( Table 1 )

**Table 1:** Distribution of FD rods.

FD rods		N = 21	Proportion (%)
Sex	Female	11	52.4%
	Male	10	47.6%
Age at rodding procedure	< 14 years	16	76.2%
	> 14 years	5	23.8%
Complications	Fracture	3	14.3%
	Loosening	3	14.3%
	Metallosis	1	4.8%
Laterality **	Right-side	10	47.6%
	Left-side	11	52.4%
Bone	Femur	18	85.7%
	Tibia	3	14.3%
Surgical indication	Deformity	4	19.0%
	Fracture	12	57.1%
	Migration	5	23.8%
Clinical Type	Type 1	4	19.0%
	Type 3	9	42.9%
	Type 4	8	38.1%

The mean age at the time of surgery was 10.47 years (minimum of 3.92 and maximum of 16.44 years). Seven (46.7%) patients underwent surgery between five and ten years of age, six (40%) patients were older than ten years, and only two (13.3%) patients underwent surgery before five years of age.

Regarding the Sillence Classification, three patients (20%) were classified as type I, seven (46.7%) as type III, and five (33.3%) as type IV. ( Table 2 )

**Table 2:** Qualitative distribution of patients according to the Sillence classification and number of FD rods.

Sillence&apos;s Clinical Type	Patients (n = 15)	Patient proportion (%)	Rods (n = 21)	Rod proportion (%)
Type I	3	20.0%	4	19.0%
Type III	7	46.7%	9	42.9%
Type IV	5	33.3%	8	38.1%

Of the 21 rods studied, 11 (52.4%) were found in female patients. With respect to laterality, eleven was on the left side (52.4%).

The main surgical indication was the occurrence of fractures (57.1%), followed by rod migration in 23.8% and correction of deformities in 19% of the cases. The mean postoperative follow-up time was 5.22 years (1.43 to 7.02 years).

Seven rods (33.3%) had postoperative complications ( Table 1 ). Three cases of peri-implant fracture (14.3%); three cases of loosening distal component (14.3%) and one case of metallosis (4.8%).

Eighteen rods were implanted in the femur (85.7%), and only three in the tibia (14.3%). Regarding age, 16 rods (76.2%) were implanted in children under 14 years of age and the remaining five (23.8%) were implanted in children over this age. For the telescoping analysis, patients were divided into these two groups in order to better assess the growth potential of younger patients. ( Table 1 )

Analyzing the data related to telescoping we found that 15 rods (71.4%) showed an increase in length ( Table 3 ), whereas the other six rods (28.6%) showed no difference between their initial and final radiographic measurements.

**Table 3:** Telescoping rate of FD rods.

Rods		N = 21	Proportion (%)	P-value
Telescoping	Yes	15	71.4%	<0.001
	No	6	28.6%	

**Figure 1:**
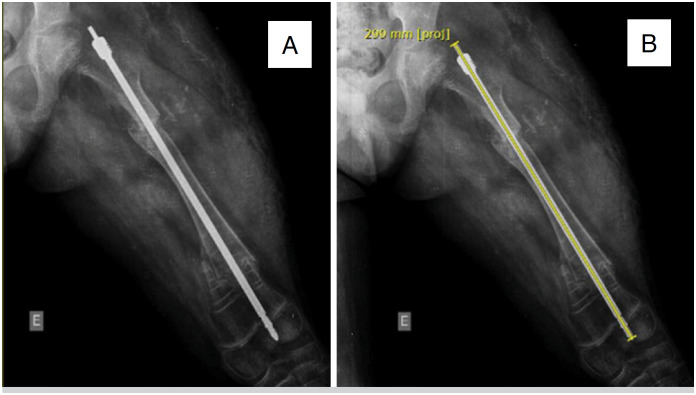
A) Anteroposterior (AP) radiograph of the left femur with fracture in its proximal and distal thirds fixed with FD rod in the immediate postoperative period (IPO). B) The same radiograph showing initial length of 299 mm in the IPO (2016).

**Figure 2:**
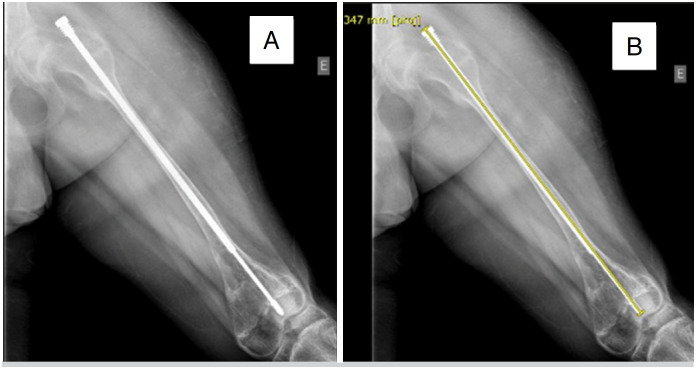
A) Anteroposterior (AP) radiograph of the left femur showing the treatment progression four years following FD rod surgery; B) The same radiograph with a final length measurement of 347mm (2020).

The overall telescoping mean was 24 mm, that corresponded to a 9.9% increase ( Figures 1 and 2 ). When considering patients who underwent surgery under 14 years of age, the mean telescoping was 30 mm (p-value<0.001) or 11.4% (263 - 293 mm). The greatest telescoping value was 71 mm, accounting for a 27.9% increase. Data from patients for whom no telescoping was observed (three rods) were disregarded. When considering patients over 14 years of age, telescoping was not statistically significant (p-value = 0.293).

According to the Sillence classification, all three clinical types showed increased radiographic measurements of Fassier-Duval rods. Type I patients had a mean of 1 **9m** m (297 - 31 **6m** m); type III had a mean of 2 **8m** m (294 - 32 **2m** m); and type IV had a mean of 2 **2m** m of lenght (272 - 29 **4m** m). A statistically significant difference was found with respect to the laterality: the mean telescoping on the left side was 35.9 mm (14.4%) versus 10.9 mm (4.9%) on the right side (p-value = 0.016). ( Table 4 )

**Table 4:** Assessment of percentage telescoping increase.

Telescoping (%)		Mean increase (%)	P-value
Sex	Female	12.2%	0.300
	Male	7.3%	
Laterality	Right-side	4.9%	0.037
	Left-side	14.4%	
Bone	Femur	8.7%	0.220
	Tibia	17.0%	
Age range	< 14 years	12.4%	0.053
	> 14 years	1.8 %	
Follow-up time interval	0.5-3 years	1.3%	0.118
	3-5 years	13.4%	
	5-10 years	12.0%	
Surgical indication	Deformity	5.1%	0.640
	Fracture	11.1%	
	Migration	10.8%	
Clinical Type	Type I	7.0%	0.788
	Type III	11.6%	
	Type IV	9.3%	

There was no statistically significant difference in telescoping with respect to the specific postoperative period. ( Table 5 ) Six patients did not show positive telescoping: three of them were over 14 years old at the time of surgery, whereas the other three, despite their young age, did not show bone growth, the reasons for which will be discussed below. ( Table 6 )

**Table 5:** Telescoping analysis of the FD rod at the initial and final postoperative time interval.

Postoperative time interval	0.5-3 years	3-5 years	5-10 years	p-value
Telescoping mean (mm)	5.2	32.2	28.5	0.142
Telescoping mean %	1.3	13.4	12	0.118

**Table 6:** Description of patients who did not show telescoping.

Age at surgery (years)	Sex	Clinical Type	Bone	Surgical indication	Postoperative follow-up time (years)	Cause of the lack of telescoping
12.3	Female	III	Femur	Fracture	5.61	No bone growth
14.22	Female	IV	Femur	Migration	6.73	No bone growth
7.77	Female	IV	Femur	Deformity	0.88	Postoperative time
6.15	Male	IV	Femur	Deformity	1.65	No bone growth
18.31	Male	III	Femur	Migration	2.52	No bone growth
15.28	Male	III	Femur	Fracture	6.59	No bone growth

## DISCUSSION

One of the main advantages of the telescopic FD rod is its ability to lengthen in synchronization with the child's bone segment. This reduces the number of revision surgeries over time and as the patient grows, thereby resulting in lower morbidity for the child with OI. Another advantage lies in the biomechanical principle of intramedullary rods in general, which work as an internal template, preventing deformities and promoting increased “resistance” against fractures due to the structure of the material itself. As disadvantages, there are difficulties in performing the percutaneous technique that includes a surgeon's long learning curve and the high cost of the material that limits its use in the public health system.^14^

Among the technical difficulties encountered in surgical procedures, the following ones can be recited: the need for multidisciplinary care, starting with the proper handling and positioning of the patient in order to prevent fractures provoked both by the nursing team and the surgical team in the room; special caution during anesthesia so as to avoid the use of anesthetics that can potentially cause hypermetabolic reactions or intubation-related mandibular and cervical fractures;^15^ and the caution and attention needed when performing the surgical technique proper, which involves inserting the intramedullary rods into very thin and commonly obliterated bones.

**Figure 3:**
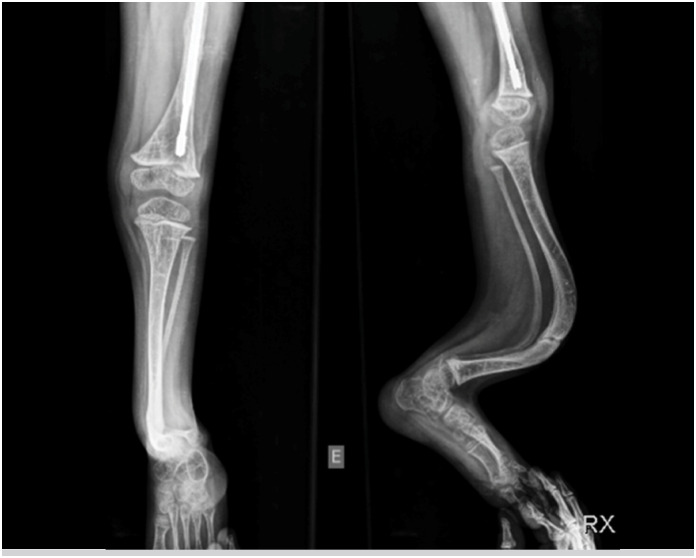
AP and lateral radiograph of the left leg showing the “rib-like” appearance of the tibia and fibula in a patient with osteogenesis imperfecta. Note the anterior convexity, with a lower bone density, compared to the posterior concavity, which is denser and more rigid, with obliterated marrow.

**Figure 4:**
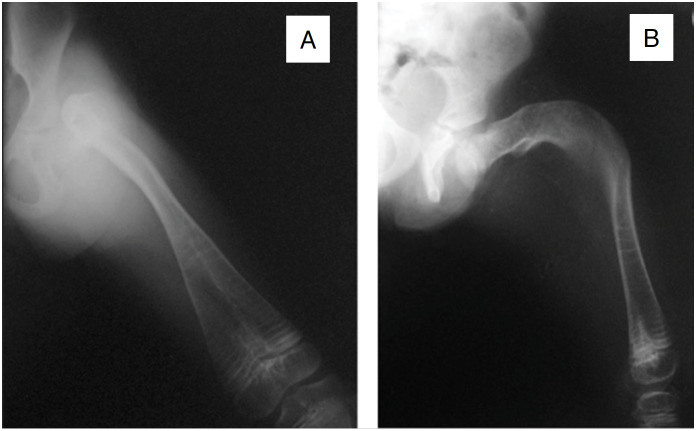
A) AP radiograph of the left thigh of a nine-year-old patient with OI and extreme varus of the proximal femur and bone overlap. B) Lateral radiograph of the same patient showing the flexion deformity.

With regard to this last aspect, it is not uncommon to find bowing deformities, as if these bones had assumed a “rib-shaped” appearance ( Figure 3 ), especially when the tibia and femur are involved. The posterior aspect (concavity) of such bones is rigid and hard, which renders it difficult to receive a reamer or guide wire, whereas on the other hand, their anterior aspect (convexity) is fragile and offers nearly no resistance, which increase the occurrence of false path of the rods. During the surgical procedure, the correction of these deformities often requires an anterior wedge-shaped shortening to better acquire a rectilinear bone pattern. In the femur another challenge is the subtrochanteric deformity that forms a proximal fragment flexed and in varus – muscle force makes intraoperative reduction difficult by forcing the rod into an anterior and lateral position, often “tearing” the bone due to the continuous muscle tension exerted. ( Figure 4 )

In Brazil, a telescopic intramedullary rod was developed by our team in 2000. Its creation was based on the principles of previous rods^7 – 12^ , but now aiming at joint preservation as well as the Fassier-Duval rods’ features. It was centrally attached into the distal epiphysis through its internal rod by means of a thread, and the trochanteric stabilization was achieved by the external rod through transosseous suturing of the greater trochanter. It yielded satisfactory results, both in terms of cost-effectiveness and complication rates.^12^ Its main limitation, though, is that its use was restricted to the femur, since only the greater trochanter allowed for proximal fixation by suturing.

The main indication of surgery with the Fassier-Duval rod in this study was the occurrence of fractures (57.1%), a result similar to that found with the rod developed by SCSP.^12^ This piece of data differs from most studies in which the main surgical indication observed was the correction of deformities.^16 , 17^ A particularity of the health care service institution where this two studies were carried out is the large volume of emergencies, making it difficult to prioritize elective surgeries, which may have influenced the indication.

Another possible bias of this study is that all patients were treated in a public hospital, at which neither the costs of FD rodding nor the procedure itself is standardized by Brazil's Ministry of Health. In the cases included in this study, the implants were obtained via donations or lawsuits.

In the seven cases (33.3%) in which there were late complications, reoperation was performed in one patient (4,8%) with removal of the FD internal component after trauma with a peri-implant fracture.The patient remained with the complete rod (two components) for 2.55 years. The other patients had conservative treatment with a plaster cast, due to the unavailability of a new FD rod. Two of them sustained perisynthesis fracture, three patients had loosened the distal tip of the FD rod, and one had metallosis. There was no relationship with the Sillence classification, given that all types included in the study presented with the aforementioned complications.

**Figure 5:**
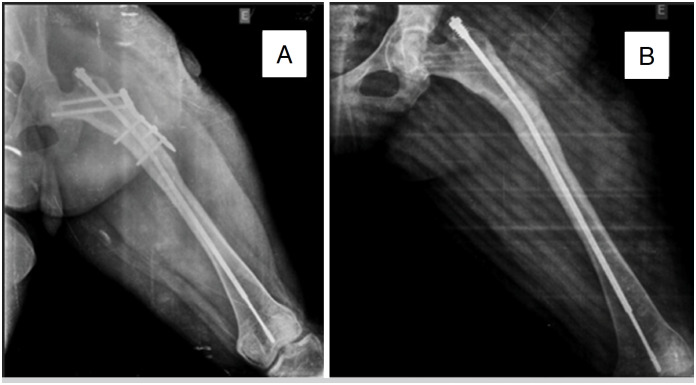
A) AP radiograph of the left femur of the patient with suspected metallosis in the proximal third of the left thigh due to likely incompatible synthesis materials. B) AP radiograph of the left femur of the same patient after removal of the plate in the IPO.

One patient had clinical signs suggestive of metallosis, but this complication is not directly related to the rod. This patient progressed to a femoral neck fracture 2 years thereafter, at which point she underwent treatment with a locking plate and due to likely incompatible materials, she started to present local symptoms of inflammation. It was opted to remove the plate. ( Figure 5 ) The problem was resolved after the implants were removed. Until the end of this study, the postoperative follow-up of this patient was 6.39 years, with the FD rod still in place and no local changes.

In this study, in addition to a longer mean follow-up time, longer than some follow-up time series found in the literature, we observed lower complication rates. Birke et al.,^4^ in a 12-month follow-up, found a complication rate 40%, and 13% of the cases required rod revision, which also happened mainly due to fractures and/or migration of one of the components. This result was similar to that found by Sulko et al.,^16^ who recorded a follow-up period of 18 months. It is worth mentioning that, in the literature, the average surgical revision rate varies between 3-14%.^3^ These data may be related to the difficulties pertaining to the surgical technique proper and the long learning curve involved in the treatment of children with fragile bones. Despite this, some of the published are much higher, like those in the study by Azzam et al.,^17^ in which 46% of the rods had to be revised, mainly due to fractures. This high rate can be explained by the greater number of patients analyzed and the long mean follow-up time (9 years).^17^ In this way, also in the series corresponding to the implant developed at SCSP, a revision rate of almost 50% was observed, mainly due to fractures.^12^

The main advantage advocated for modern rods is their ability to telescope immature skeletons, thereby reducing the number of surgical reinterventions.^6,7,12,^ In this study, 71.4% of the rods showed positive telescoping, with an average increase of 24 mm (9.9%) relative to the initial size. Considering patients under 14 years of age, the mean absolute lengthening achieved with telescoping was 30 mm, corresponding to an 11.4% increase. In the series recorded with the rod developed at SCSP, we observed positive telescoping in 60% of the rods, similarly to what was found in the current study. In the previous study with the SCSP rod, the mean increase was 23.57% – in absolute figures, the mean increase was 48.3 mm.^12^ These results are probably attributable to the longer follow-up time (about nine years versus five years). In the literature, when telescoping failure is observed, the results are similar, however such studies do not present quantitative data, either as percentages or absolute values, that might allow for a comparative analysis.^4 , 12 , 18^ Both the technical difficulty in assessing size and proportionality with the software used to view the radiographs by different health care services and the lack of parameter standardization when taking the measurements of the rods during the follow-up period make the analysis of these data difficult. We hope that further studies can provide advances in ensuring the reliability of this important piece of data on the main feature of telescopic rods in the future.

Of the six patients who did not show positive telescoping, four had reached skeletal maturity at the time they underwent surgery, one with 12.3-year-old female patient and three others who were older than 14 years of age. The other two patients were under 14 years of age and did not show bone growth, whereas the postoperative time was insufficient for growth analysis. ( Table 5 )

In the literature, the main cause indicated for telescoping failure is internal component migration of the FD rod (with distal fixation), with rates ranging from 13% to 41%.^4 , 12 , 13^ In this study, the main reason for the lack of telescoping was the absence of bone growth among the patients. Lack of telescoping was also seen in a child with less than 1 year follow-up and one 6.15-year-old patient, despite a follow-up of 1.65 years, did not show bone growth in the period when comparing the radiographs.

Despite the good results found in both this study and the previous one carried out by our work group, our biggest limitation is the small series (15 patients in the current study and 22 patients in the previous one), given that osteogenesis imperfecta is a rare disease.

## CONCLUSION

We found good effectiveness of surgical treatment in OI patients with the FD rod, with telescoping success rates like those reported in the literature, and with our rods^12^ , as well as similar complication rates.^4 , 12 , 18^

Other studies with specific data on telescoping, i.e., absolute values and percentages, are still scarce, which does not allow for a better comparison of either growth parameters or results.
